# Customer Orientation and Leadership in the Health Service Sector: The Role of Workplace Social Support

**DOI:** 10.3389/fpsyg.2017.01920

**Published:** 2017-11-01

**Authors:** Andreina Bruno, Giuseppina Dell’Aversana, Anna Zunino

**Affiliations:** Department of Education Sciences, University of Genoa, Genoa, Italy

**Keywords:** customer orientation, health service, leadership, workplace social support, task-oriented behavior, relationship-oriented behavior, patient-centered care

## Abstract

Health care is a critical context due to unpredictable situations, demanding clients, workload, and intrinsic organizational complexity. One key to improve the quality of health services is connected to the shift in organization perspective of viewing patients as active consumers rather than passive users. Therefore, higher levels of customer orientation (CO) are expected to improve organizational service effectiveness. According to a cultural perspective to CO, the aim of the study was to explore how different leaders’ behaviors (task-oriented and relationship-oriented) interact with CO of health organizations. Specifically, the aim of the paper was to contribute to this topic, by considering the leaders’ point of view. Since leader’s experience of CO is influenced by social processes in the work environment, workplace social support (WSS) was inserted as moderator in the relationship between leader behavior and CO. A survey study was conducted among 57 Health Department directors belonging to the National Health Service in the North of Italy in 2016. Findings showed that WSS moderated the influence of leadership concern for relationship on CO. Practical implications of the study are discussed.

## Introduction

Health care is a critical context due to unpredictable situations, demanding clients, workload, and intrinsic organizational complexity. The need for health-care quality improvement in a period of increasing financial and service pressures requires to not separate financial performance and productivity from service quality. The King’s Fund (2013) points out that the quality of care provided by health organizations is a corporate responsibility: “Boards should be held to account for ensuring that their organizations achieve high standards of patient care, and that serial failures do not occur” (p. 9). The Institute of Medicine (IOM, 2001) report articulates six critical aims for health-care system. Care must be delivered by systems that are carefully and consciously designed to provide care that is safe, effective, patient-centered, timely, efficient, and equitable.

One key to improve the quality of health services is connected to the shift in organizations’ perspective of viewing customers as active co-producers rather than users. Aiming at patient-centered care, hospital business is required to treat patients as customers. By considering customers as the first priority, not only client and staff satisfaction significantly increases as positive relationships with patients act as protective factors and lessen social stressors (Guglielmetti et al., 2014), but also clinical care outcomes improve (Zablah et al., 2012; Rania et al., 2015), following the double empowerment effect between customers and workers that has been highlighted in previous studies (Converso et al., 2015). Customer orientation (CO) predicts several important customer outcomes, such as customer-perceived service quality and satisfaction (Susskind et al., 2003), indicating that health providers’ delivering service on the front lines have an influence on patients’ experience with services. Moreover, leadership has a primary role in supporting organizational CO (Liao and Subramony, 2008). In the next sections, we described the concept of CO and the role of leadership in relation to it.

### Customer Orientation

The term CO means the focus on meeting customers’ interests, needs, and expectations, and on delivering appropriate and personalized services. In the case of the health-care sector, where patients are the customers, it is defined as the ability of service providers to adjust their service, in a way that reflects patients’ reality (Daniel and Darby, 1997).

The concept has been approached in two distinct ways. The first one considers CO as a personal attitude or a surface-level personality trait, which refers to “an employee’s tendency or predisposition to meet customer needs in an on-the-job context” (Brown et al., 2002, p. 111) and as an antecedent of job outcomes as job satisfaction and job performance (Donavan et al., 2004; Matthews et al., 2016; Miao and Wang, 2016).

The second one considers CO as a set of organizational behaviors (Saxe and Weitz, 1982) connected to the organizational culture (Narver and Slater, 1990). CO is a service practice that assesses “the degree to which an organization emphasizes, in multiple ways, meeting customer needs and expectations for service quality” (Schneider et al., 1998, p. 153). It is embedded within the marketing concept of the health organization and promotes the dissemination of market-related knowledge, enabling the organization to deliver high quality care in a consistent and immediate manner (Hallums, 2008). Following Normann (1984), this approach permits to recognize the impact on the service quality of the quality of the relationship between customer and provider as well as between provider and leader. Indeed, the two different relationships are strictly interrelated: the quality of relationship is like a cascade flow from the back-office (or the top) to the front-line of the service process. In this way, although being patient-focused is essential, effective CO presupposes also to consider internal customers. “Because internal customers (employees) provide services to external customers (patients), their role is vital for delivering care of high quality and satisfying patients. […] employees will be willing to do their best in order to satisfy the needs of patients only after effective internal exchanges at their level have taken place. For this reason, unless an organization focuses on internal operational excellence, other than the market, continuous achievement and organizational effectiveness cannot be achieved” (Bellou, 2010, p. 386).

Most of the studies on CO conclude their discussion with managerial implications. All of them recommend formal leaders to implement specific organizational strategies to improve CO. In the next section, we explored the managerial issues related to CO. Following McVicar (2016), we split managerial issues into leadership behaviors (Blake and Mouton, 1964, 1982; Pruitt and Rubin, 1986) and workplace social support (WSS; Hobfoll, 2002).

### Leadership Behaviors and Customer Orientation

Leadership is considered one of the most important determinants on organizational processes: the quality of leadership has been linked to a multitude of outcomes within occupational health psychology. There is ample evidence suggesting that leaders play a key role in influencing employee attitudes toward customers (Liao and Subramony, 2008). “Managerial philosophies and values influence organization’s internal business practices, which, in turn, influence employee and customer interactions and behaviors” (Susskind et al., 2003, p. 180). In the medical literature, effective leaders, from executives to front-line managers, have been shown to contribute to the implementation of an organizational culture that values quality of care. Leaders can encourage care quality by promoting CO (Gountas and Gountas, 2016), patient-centered care, rather than provider-centered (Pelzang, 2010; Cliff, 2012; Dell’Aversana and Bruno, 2017) and patient safety (Kaplan et al., 2010; Bruno and Bracco, 2016). As Shaller (2007) pointed out, the most important factor contributing to health-care improvement is the commitment and engagement of senior leadership. Specifically, leadership has a crucial function since it determines the quality of relationship within the team, thus influencing the quality between employees and clients. Sustaining the quality of relationship within the team (internal clients) improves the quality of relationship with the external clients, thus permitting to increase the quality of health services (Corrigan et al., 2000; Kim and Lee, 2016).

Reviews covering decades of leadership research agree on two predominant types of leadership behavior: relations-oriented behavior and task-oriented behavior (Judge et al., 2004). Differences in leaders’ performance can be explained by the extent to which the leader is task- or person-oriented (Yukl et al., 2002; Kellett et al., 2006; Gaubatz and Ensminger, 2017). In particular, Stock and Hoyer (2002) found positive relationships between employees’ CO and different leadership styles: leaders’ emphasis on task achievement, leaders’ supportiveness, and initiation of CO. Moreover, there is evidence that leader’s CO predicts employee’s CO (Strong, 2006).

Although leadership behaviors appear as a crucial factor for promoting a culture that focuses on customer relationships and customer service (Anaza et al., 2016), according to Liaw et al. (2010), “it is somewhat surprising that the ways in which leadership behaviors influence customer orientation have received little attention in the existing literature” (p. 478). Indeed, in previous studies, researchers have attempted to identify the antecedents of CO by examining predominantly the main effects of leadership on CO employees’ perceptions. Compared with the large literature on employees, less attention has been paid on CO for organizational leaders (Schuh et al., 2012).

### Workplace Social Support

Workplace Social Support (WSS) is a key construct implicated with a variety of health and organizational outcomes: it is a resource in the work environment which can be employed in dealing with complex problems (De Jonge et al., 2008).

More specifically, in the cultural perspective of CO expressive emotional network resources are recognized as drivers of CO, as form of social capital that provides direct access to information and emotional support to improve CO (Anaza et al., 2016). WSS may increase performance in two ways: “first, it provides individuals with emotional support. Second, having a network for emotional support among coworkers helps employees maintain the required patience, care, and focus needed to provide high-quality customer service even in the face of challenging interactions. Coworker support provides greater job resources to deal with stressful and difficult customers” (Anaza et al., 2016, p. 1475). In this way, WSS plays a role in creating value for customers. Walsh et al. (2015) claimed that CO is affected by WSS, finding that an empowered work environment enhances employees’ affective responses and these affective responses should spillover to the employee–customer interface, leading to a greater level of CO.

The same process has not yet been explored for leaders. Tepper and Taylor (2003) argued that supervisors who perceived they were treated fairly by the organization could reciprocate by treating subordinates more favorably. Expressive emotional network resources may support leaders in their middle position between customers’ service excellence demands on one hand, and productivity and performance requirements on the other hand (Babakus et al., 2009; Cortini, 2016). Indeed, there is evidence on the different way that managerial and non-managerial employees perceive organizational values (Schneider et al., 1998) and on the different extent to which they focus on customers’ needs (Martin and Fraser, 2002).

According to the cultural perspective to CO, we expect that leader’s experience of external CO is influenced by social processes in the workplace. Therefore, in our study, the focus is on leaders. Focusing on formal leaders does not imply that leadership as social influence is limited to these roles, but means that those in formal leadership roles have a particularly strong potential to affect outcomes relevant to organizations, especially the organizational culture, that in turns influences the quality of care (Kaissi et al., 2004).

## Aims

In light of the previous theoretical constructs, the aim of this study was to analyze leaders’ perceived CO in relation to leadership behavior and WSS in the health-care service context.

More specifically, we explored (a) the different ways leaders’ behavior (task-oriented and/or relationship-oriented) is related to a wider or lesser CO and (b) if the WSS moderates this relationship.

## Method

A survey study was conducted among 62 Health Department directors belonging to the National Health Service in the North of Italy in 2016. All the directors were attending a course of managerial competences revalidation.

Participants were given an anonymous questionnaire, of whom 57 were usable. All subjects gave written informed consent and authorized and approved the use of anonymous/collective data for publications. Given the masculine dominant nature of our sample, we did not consider gender as a control variable.

In line with recent works (Thomas et al., 2001; Anaza et al., 2016), the CO was measured through the SOCO Scale (Saxe and Weitz, 1982). We used the 24-item, 9-point version revisited by Hoffman and Ingram (1992) in order to fit the health-care sample (α = 0.89). Examples of items are as follows: “I try to help patients achieve their goal”; “I try to achieve my goal by satisfying patients.”

In relation to leadership, the two dimensions ‘concern for task’ and ‘concern for relationship’ were measured through Blake and Mouton’s (1982) Managerial Grid, on which research and theory on leadership converges and the Dual Concern Theory is based (Pruitt and Rubin, 1986; Kellett et al., 2006; Garg and Jain, 2013). We used eight items of the subscale ‘Concern for task’ (α = 0.58) and eight items of the subscale ‘Concern for relationship’(α = 0.69), scored on a 5-point rating scale. Sample items are as follows: “Nothing is more important than accomplishing a goal or task” for ‘Concern for task’; “I encourage my team to participate when it comes decision making time and I try to implement their ideas and suggestions” for ‘Concern for relationship.’

Workplace social support (WSS) was measured through six items of the Demand-Induced Strain Compensation Questionnaire in its 5-point scale, Italian version (Bova et al., 2015) (α = 0.74). Sample items include: “Employee X will feel esteemed at work by others.”

## Findings

### Preliminary Analysis

Descriptive statistics (mean and standard deviation) and correlation of research variables are shown in **Table 1**. Significant positive relationships are shown between leadership concern for relationship, CO, and WSS, whereas leadership for task correlates positively with leadership concern for relationship. To address the common method bias, we adopted the Harman’s single-factor test (Conway and Lance, 2010).

**Table 1:** Correlations (Pearson’s *r*) and descriptive statistics of the scales (*N* = 57).

	1	2	3	4
(1) Leadership ‘Concern for relationship’				
(2) Leadership ‘Concern for task’	0.447^∗∗^			
(3) Workplace social support	0.328^∗∗^	0.185		
(4) Customer orientation	0.242^∗^	0.215	0.290^∗^	
Mean	4.04	3.85	3.3	7.6
Standard deviation	0.41	0.40	0.57	0.80
*N* item	8	8	6	24

*^∗^*p* < 0.05, ^∗∗^*p* < 0.01.*

### Moderation Analysis

Two moderated regression analyses were used to test the moderating role of the WSS on the relationship between leadership behavior (‘concern for task’ and ‘concern for relationship’) and CO. We used PROCESS macro for SPSS developed by Hayes (2013), using model n. 1. In the first analysis, we had leadership concern for relationship as independent variable, the WSS as moderator, and CO as dependent variable. In the second one, we tested leadership concern for task as independent variable with the same moderator and dependent variables. The variables were mean-centered prior to analysis.

#### Leadership ‘Concern for Relationship’

**FIGURE 1:**
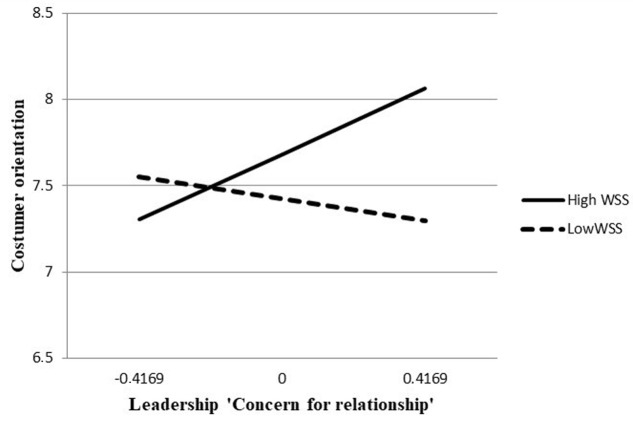
Moderation analysis.

Regression analysis found there was no main effect of leadership concern for relationship on CO [*b* = 0.30, *SE* = 0.25, *t*(53) = 1.21, *p* = 0.2294] and of WSS on CO [*b* = 0.22, *SE* = 0.18, *t*(53) = 1.23, *p* = 0.2224]. Most importantly, we obtained the interaction between leadership concern for relationship and WSS on CO [*b* = 1.05, *SE* = 0.37, *t*(53) = 2.83, *p* = 0.0065]. As shown in **Figure 1**, analyses revealed that leadership concern for relationship was positively related with CO for higher levels of WSS, *b* = 90, *SE* = 0.32, *t*(53) = 2.80, *p* = 0.0070. Conversely, the relationship between leadership concern for relationship and CO was not significant for lower levels of WSS, *b* = -30, *SE* = 0.33, *t*(53) = -0.92, *p* = 0.3615 (**Figure 1**).

#### Leadership ‘Concern for Task’

Analyses revealed that there was no main effect of leadership concern for task on CO, *b* = 0.35, *SE* = 0.26, *t*(53) = 1.37, *p* = 0.1758 and of WSS on CO, *b* = 0.20, *SE* = 0.20, *t*(53) = 1.02, *p* = 0.3107. Moreover, analyses revealed no significant interaction between leadership concern for task and WSS on CO, *b* = 0.96, *SE* = 0.50, *t*(53) = 1.92, *p* = 0.0605.

## Discussion, Conclusion, and Practical Implications

Due to the role of the health service culture in the development of service quality and customer perceptions of organizational image (Senge, 2006), there is a need to study the factors that affect the quality of CO, including leaders’ behavior and WSS. Our study contributes to the debate analyzing the different ways leaders’ behavior is related to a wider or lesser CO and if WSS moderates this relationship.

Findings did not show any direct effect of leadership behaviors on CO. This effect was moderated by the presence of higher level of WSS. Findings showed that the abovementioned resources moderated the relationship between leadership concern for relationship and CO. No moderating effect was found for the relationship between leadership concern for task and CO. However, this finding could be affected by the poor reliability of the leadership ‘concern for task’ scale.

Findings highlight the important role of WSS in stimulating health-relevant aspects of leadership behavior (Gregersen et al., 2016). In fact, the leadership dimension ‘concern for relationship’ seems to be related to CO, only if leaders can refer to higher resources in their work environment. Undoubtedly, listening to information from the front-line, sharing information, taking care of the quality of relationship with and among their collaborators, monitoring their identification in organizational values and patients’ needs, using feedback is high-demanding. One key point emerging from this study is that providing health-care contexts with higher expressive emotional network resources could facilitate leadership focusing both internal and external clients (Cortini et al., 2016).

A second key point of the study is the different roles of leaders’ behaviors. ‘Concern for task’, i.e., the focus on task and goals achievement, is traditionally considered the primary dimension of leadership in the bureaucratic model, in which standardization of procedures is the answer to the complexity of organizational processes as well as to the need for transparency of Public Administrations.

On the contrary, service organizations need to take into account the variability of the clients and the need for personalization of services. The diversity of each client, inputting variance and risk of fragmentation into the system, requires leaders to protect integrative functions. This study supports the need for health services to interpret leaders’ role as not merely oriented to accomplishment of tasks and focus on goal, standards, and performance. In this direction, leadership means maintaining or improving processes that facilitate accomplishment of tasks not only by clarifying role expectations and standards for task performance, but also by caring for their collaborators. “Relations-behaviors largely concern maintaining or improving cooperative interpersonal relationships that build trust and loyalty. Relations-behaviors include listening carefully to others to understand their concerns, providing support and encouragement, helping, and recognizing people as individuals” (Kellett et al., 2006, p. 150).

Our findings offer several implications. First, if the health system wants to increase its patient-centeredness, it must take care of its leaders, by providing them with resources in their job context, and more specifically social support resources. For instance, leaders have to be accompanied through recruitment and training to make their managerial competencies related to concern for relationship more visible, accessible, and reflective (Bruno and Dell’Aversana, 2017a,b,c). In Italy, this issue is quite relevant, since in health domain directors are primarily chosen on their technical skills. On the contrary, their leadership competencies are generally taken for granted, and not always evaluated or trained before and during their work experience as leaders.

A further implication is based on the acknowledgment that the quality of relationship in the back-office may have an important effect on the quality and degree of CO. Hence, the study extends work that has shown the relationship between internal and external marketing (Kim and Lee, 2016). Organizations need to provide devices to focus on internal marketing in order to sustain CO.

## Limitations

There are some limitations to the present research. The main limitations are the small sample size, the cross-sectional design of the study, and its reliance on self-report measures. Future longitudinal studies should investigate other causal directions or even reciprocal relations of the variables more profoundly. Second, the study used single-source data. Future research may overcome this limitation by collecting data from multiple sources, for instance, customer assessments of health service and collaborators’ evaluation of leadership behavior. We have not addressed the relationship of CO and traditional performance measures. Future research is necessary to integrate these outcomes.

## Ethics Statement

The questionnaire included a statement regarding the personal data treatment, in accordance with the Italian privacy law (Law Decree DL-196/2003). The workers authorized and approved the use of anonymous/collective data for scientific publications. Because the data were collected anonymously and the research investigated psychosocial variables not adopting a medical perspective, ethical approval was not sought.

## Author Contributions

AB, GDA, and AZ conceptualized the study and the theoretical framework. AB wrote the Section “Introduction.” AB and AZ collected data. GDA analyzed the data and wrote the Sections “Method” and “Findings.” AB wrote the Section “Discussion, Conclusion, and Practical Implications.” All the authors then revised and improved the manuscript several times.

## Conflict of Interest Statement

The authors declare that the research was conducted in the absence of any commercial or financial relationships that could be construed as a potential conflict of interest.
